# Epidemiologic sequential analysis of pure red blood cell aplasia and T-cell large granular lymphocyte leukemia in Korea

**DOI:** 10.1007/s00277-025-06406-x

**Published:** 2025-05-22

**Authors:** Sooyong Park, Hyun Kyung Kim

**Affiliations:** 1 Department of Laboratory Medicine, Chungnam National University Hospital, Daejeon, Republic of Korea; 2 Department of Laboratory Medicine and Cancer Research Institute, Seoul National University College of Medicine, Seoul, Republic of Korea

**Keywords:** Pure red cell aplasia, T-cell large granular lymphocyte leukemia, Big data, National health insurance service, Epidemiology

## Abstract

Pure red cell aplasia (PRCA) is a rare hematologic syndrome characterized by anemia with marked reticulocytopenia and, in Asia, is often accompanied by T-cell large granular lymphocyte leukemia (T-LGL). Minimal research has been done on the epidemiology and sequential events of PRCA combined with T-LGL. This study identified 2801 PRCA and 840 T-LGL patients by using big data of the National Health Insurance Service between 2003 and 2022. The average annual crude incidence of PRCA was 2.77 per million and remained stable over 20 years, while T-LGL incidence was 0.82 per million with an increasing trend, possibly reflecting improved diagnostic accessibility. The average age for PRCA and T-LGL onset increased over the study period, consistent with aged society. Associated PRCA conditions are rheumatic diseases (10.5%), thymoma (4.7%), parvovirus infection (1.0%), inflammatory bowel diseases (0.8%), T-LGL (0.6%) and no specific cause (82.4%). Among 18 patients with both PRCA and T-LGL, PRCA preceded T-LGL (50%) or diagnosed concurrently (44%), suggesting that autoreactive T cells in PRCA which suppress erythropoiesis and sequentially evolve into clonal T cell proliferation and, eventually, T-LGL occurrence. This observation supports the hypothesis that both conditions might share a common pathogenic pathway. Further study should identify the causal relationship of PRCA diagnosis followed by T-LGL diagnosis.

**Supplementary Information:**

The online version contains supplementary material available at 10.1007/s00277-025-06406-x.

## Introduction

Pure red cell aplasia (PRCA) is a rare hematologic syndrome characterized by normocytic normochromic anemia with marked reticulocytopenia due to decreased erythropoiesis. Common conditions associated with PRCA include autoimmune disorders, lymphoproliferative disorders, neoplasms and infections [1, 2]. Minimal research has been done on PRCA epidemiology. A recent Japanese study reported average incidence was 1.06 per million per year [1]. Just only through short-term data analysis from the early 2000s, a Korean study reported an average incidence was 5.28 per million [3]. Thus, analysis of long-term nationwide data is necessary.

In Asian countries, PRCA is often accompanied by T-LGL [4–6]. Our previous study reported that PRCA combined with T-LGL revealed unique characteristics such as advanced age and low VAF levels of *STAT3* mutation, which differed from T-LGL without PRCA [7]. In cases of PRCA combined with T-LGL, it is unclear which disease is considered the cause and which is the consequence, much like the “chicken and egg conundrum” [5]. We are not aware of any previous report about the temporal sequence of PRCA and T-LGL onset. Sequential understanding of events surrounding the combination of PRCA and T-LGL may provide insight into the pathogenic association of the two diseases.

This study investigated PRCA and T-LGL epidemiology in Korea using National Health Insurance Service (NHIS)-National Health Information Database (NHID) information over a 20 year period. Notably, sequential occurrence of these diseases was explored in patients with PRCA combined with T-LGL.

## Materials and methods

### Study database

The NHIS-NHID is a database covering 97% of the Korean population, where an individual’s lifelong medical history is managed by the assignment of an anonymized individual code. NHIS-NHID data from patients with PRCA (D60) and T-LGL (C91.7) were extracted based on International Classification of Diseases, 10th revision (ICD-10) codes, and date of diagnosis was considered as the date of diagnostic code was first assigned. After extracting data from 2002 to 2022, set 2002 as the wash-out period and only analyzed data from 2003 to 2022. Patients with congenital PRCA (Diamond-Blackfan anemia) and those under the age of 20 were not included to account for congenital effects. Crude annual incidence was calculated by the number of patients per million for each year. Data for associated PRCA conditions were extracted for patients with inflammatory bowel diseases (K50, K51), malignant neoplasm of thymus or thymoma (C37), parvovirus infection (B34.3), and rheumatic diseases (M05, M06, M32, M35). Region codes were extracted by residence code and state/city code. For each year of the study period, the number of general population and medicine specialists by state/city were extracted from the Korean Statistical Information Service (KOSIS). The correlation between the number of medicine specialists and the number of patients across each region was then analyzed.

### Statistical analysis

Excel Office 2019 (Microsoft, United States) and R studio version 4.3.3 (Posit, United States) were used to provide statistical analysis. The mean difference between the PRCA and T-LGL was considered statistically significant (*p* < 0.05) using the Wilcoxon signed-rank test. The Pearson correlation was used to compare the two groups.

## Results

### Epidemiology

**Fig. 1:**
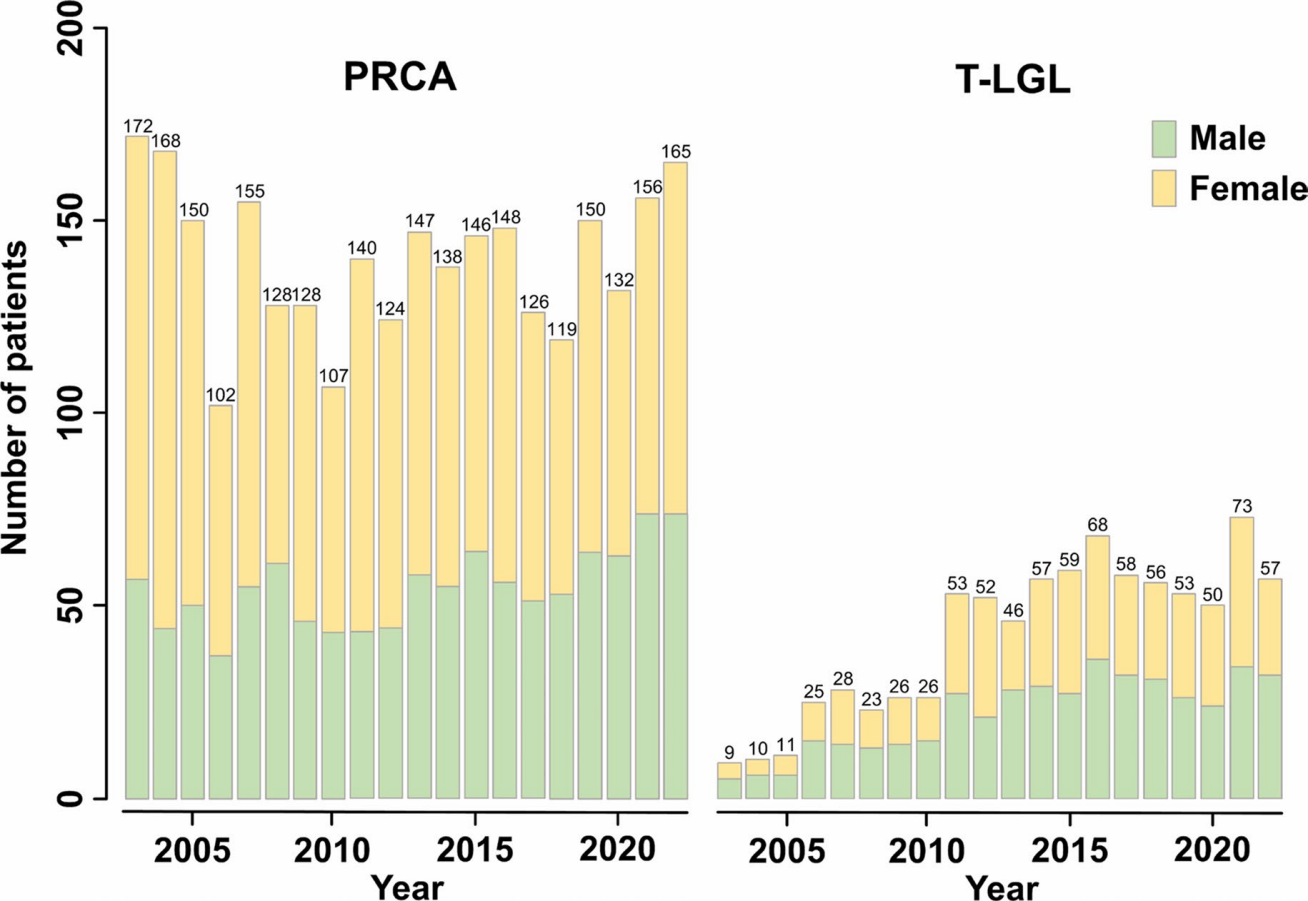
The number of patients diagnosed with PRCA and T-LGL. The number of PRCA has not changed significantly over 20 years, with a median of 143 patients per year. In contrast, the number of T-LGL more than doubled from 2011–2022 compared to 2003–2010. PRCA tended to be more common in female, but the relative proportion of male has gradually increased in recent years. In T-LGL, the difference in proportions between male and female has not changed. Abbreviations: PRCA, pure red cell aplasia; T-LGL, T-cell large granular lymphocyte leukemia

According to data from the NHIS-NHID of Korea, between January 1, 2003 and December 31, 2022, a total of 2801 patients were diagnosed with PRCA and 840 patients were diagnosed with T-LGL (Fig. 1). The annual crude incidence of PRCA in 2003–2022 was 2.08–3.53 per million and the average annual incidence was 2.77 per million (Supplementary Table 1). The highest incidence was 3.53 in 2003, and the second highest was 3.46 in 2004. During the same period, the annual crude incidence of T-LGL was 0.19–1.41 and the average annual incidence was 0.82 (Supplementary Table 2). From 2003 to 2010, the annual incidence was 0.19–0.57. However, annual incidence more than doubled between 2011 and 2022 to 0.90–1.41.

From 2003 to 2012, the median female-to-male ratio of PRCA was 1.82 (interquartile range, IQR 1.76–2.01), but significantly decreased to 1.31 (IQR 1.23–1.50) from 2013 to 2022 (*p* = 0.005). For T-LGL, the ratio was 0.82 (IQR 0.74–0.94) from 2003 to 2012 and 0.93 (IQR 0.81–1.07) from 2013 to 2022, with no significant change (*p* = 0.273).

**Fig. 2:**
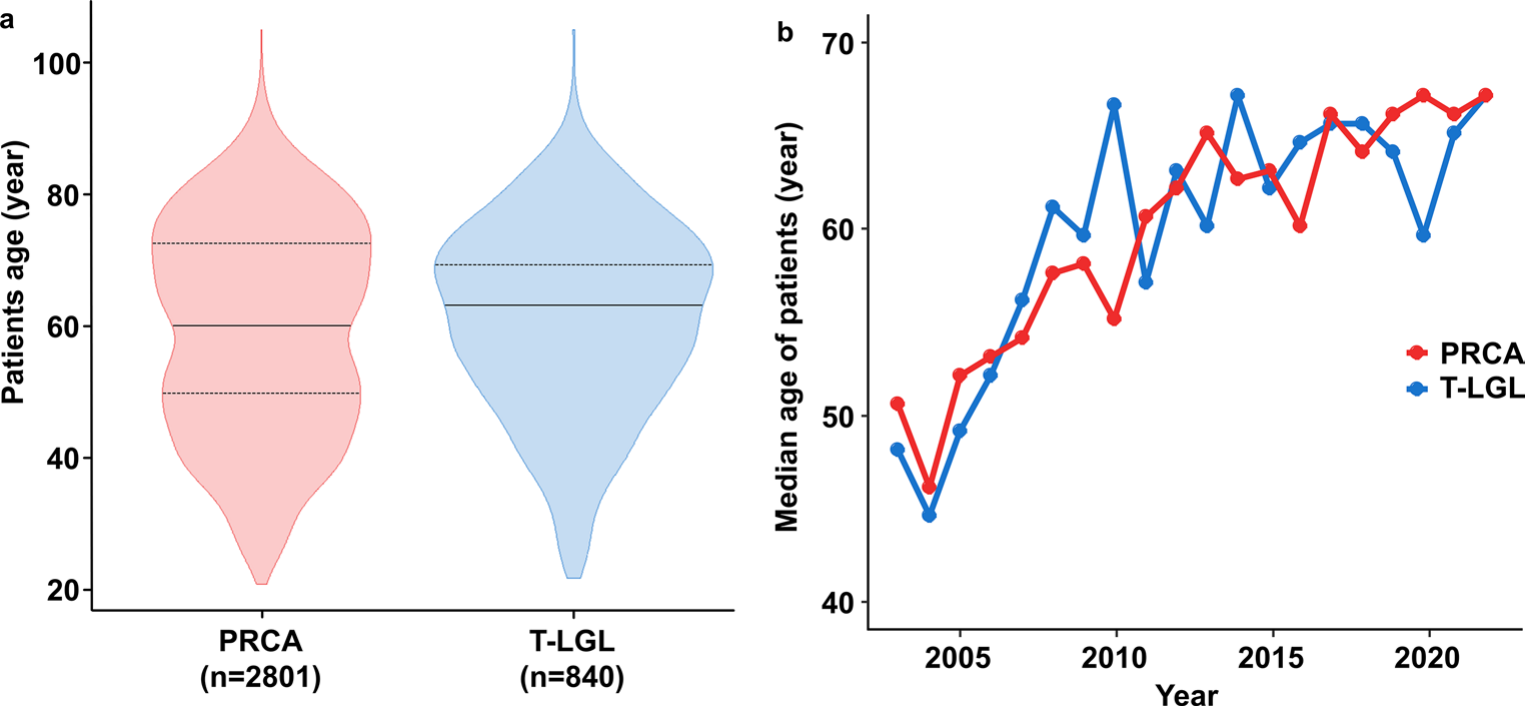
Patients age of PRCA and T-LGL at time of diagnosis. (**a**) The median age of the PRCA was 60 years (IQR 47–73) was significantly lower than that of T-LGL, which was 63 years (IQR 52–72) (*p* = 0.005). PRCA had two peaks age distribution, with lower peak at age 49.7 and higher peak at age 72.5. T-LGL had one peaks age 69.0. (**b**) The median age at diagnosis of PRCA increased from 45–58 years in 2003–2010 to 60–67 years in 2011–2022. A similar trend was seen in T-LGL. Abbreviations: PRCA, pure red cell aplasia; T-LGL, T-cell large granular lymphocyte leukemia; IQR, interquartile range

The median age of PRCA was 60 years (IQR 47–73), which was significantly lower than T-LGL, which was 63 years (IQR 52–72) (*p* = 0.005) (Fig. 2a). PRCA had two age distribution peaks, with a lower peak at age 49.7 and a higher peak at age 72.5. T-LGL had one peak at age 69.0. The most common age range for both PRCA and T-LGL was 70–79 years. For both diseases, age at diagnosis has been increasing in recent years (Fig. 2b), with the median age of PRCA increasing from 45 to 58 years in 2003–2010 to 60–67 years in 2011–2022.

### Associated PRCA conditions

**Fig. 3:**
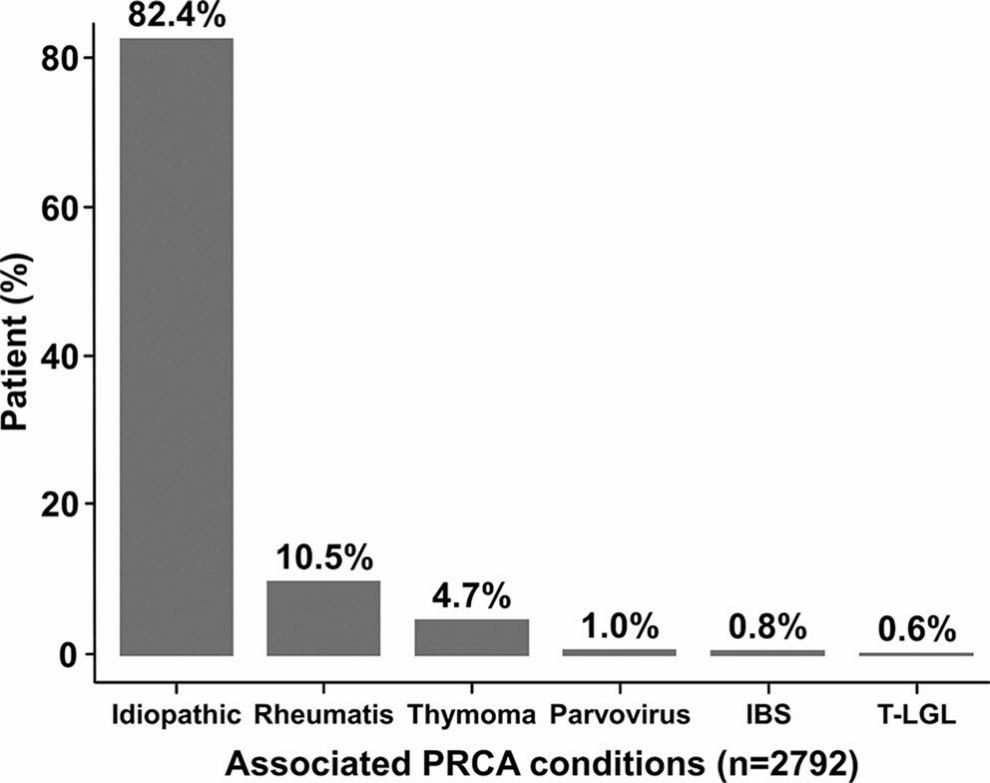
Associated PRCA conditions were rheumatis (*n* = 294), thymoma (*n* = 132), parvovirus infection (*n* = 27), IBS (*n* = 21), T-LGL (*n* = 18) and no specific cause (*n* = 2318). Abbreviations: PRCA, pure red cell aplasia; Rheumatis, rheumatic diseases; IBS, inflammatory bowel diseases; T-LGL, T-cell large granular lymphocyte leukemia

Excluding the nine follow-up missing patients, associated PRCA conditions were rheumatic diseases (10.5%, *n* = 294), thymoma (4.7%, *n* = 132), parvovirus infection (1.0%, *n* = 27), inflammatory bowel diseases (0.8%, *n* = 21), T-LGL (0.6%, *n* = 18) and non-specific causes (82.4%, *n* = 2300) supposed to be idiopathic (Fig. 3). Rheumatic diseases, including rheumatoid arthritis, systemic lupus erythematosus, and Behcet’s disease, were commonly observed as comorbidities.

### Sequential events of PRCA combined with T-LGL

**Fig. 4:**
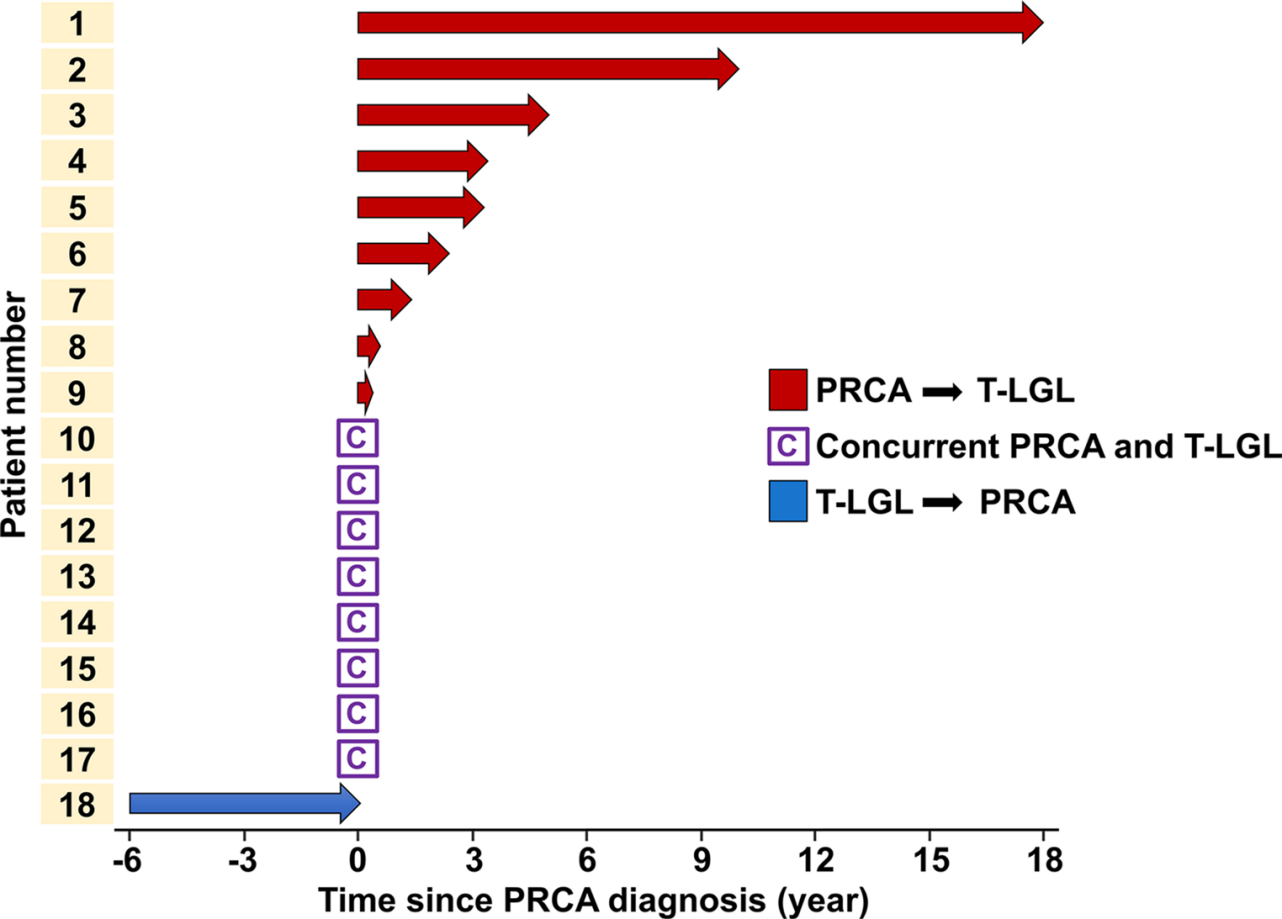
Timeline of diagnostic events in patients diagnosed with PRCA and T-LGL (*n* = 18). In nine (50.0%) cases, PRCA was diagnosed first followed by T-LGL with a median of 3.3 years later (red arrow bar). In eight (44.4%) cases, both diseases were concurrently diagnosed within a month of each other. One (5.6%) patient was diagnosed with T-LGL first, followed by PRCA (blue arrow bar). Abbreviations: PRCA, pure red cell aplasia; T-LGL, T-cell large granular leukemia

Among 840 patients with T-LGL, a total of 18 patients were identified with PRCA diagnosis. The sequential diagnostic events of these patients were investigated (Fig. 4). In nine patients (50.0%), PRCA was the primary diagnosis, followed by T-LGL at a median time interval of 3.3 years later. Median age at PRCA diagnosis was 56 years (IQR 47–66). The longest diagnostic interval between the two conditions was 18 years. In eight (44.4%) cases, both diseases were diagnosed within a month of each other and the median age at diagnosis was 60 years (IQR 57.5–73.5). Only one (5.6%) patient was diagnosed with T-LGL first with a subsequent PRCA diagnosis 6 years later.

### Regional difference

**Fig. 5:**
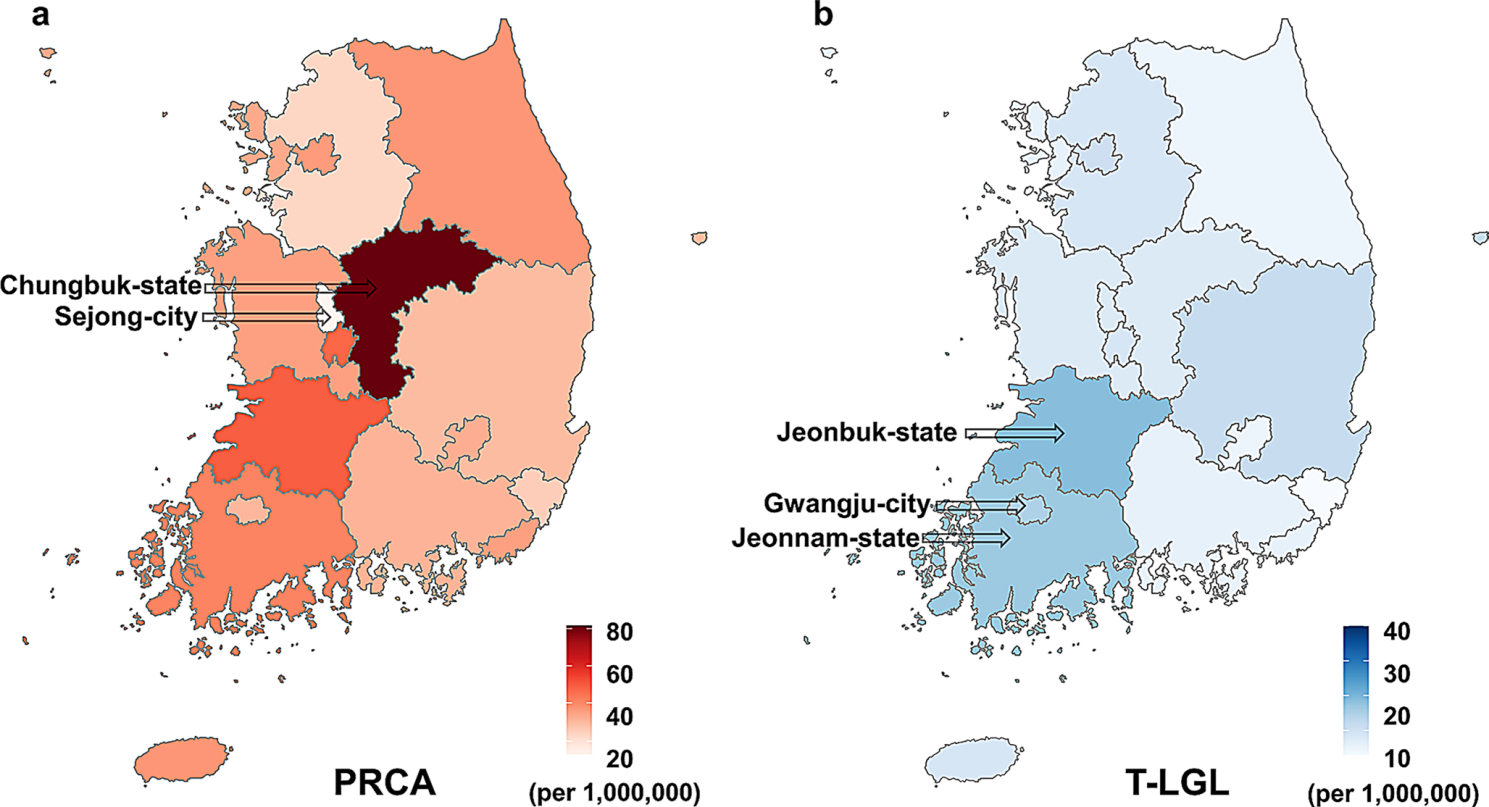
Prevalence of PRCA and T-LGL by region in Korea (at December 31, 2022). (**a**) Prevalence of PRCA was highest in Chungbuk-state (81.5 per million) and lowest in Sejong-city (13.03). (**b**) T-LGL was high in Jeonbuk-state (18.65), Jeonnam-state (16.50) and Gwangju-city (15.37). Abbreviations: PRCA, pure red cell aplasia; T-LGL, T-cell large granular leukemia

Prevalence of PRCA was 34.27 per million and T-LGL was 9.29 per million in Korea as of December 31, 2022 (Supplementary Table 3). Prevalence by region revealed PRCA was highest in Chungbuk-state (81.5), and lowest in Sejong-city (13.03) (Fig. 5a). The average annual incidence of PRCA in 2011–2016 was 9.72 per million in Chungbuk-state, which was higher than 2.74 per million in Korea, and decreased to 5.74 per million in 2017–2022, but it continues to show a high trend (Supplementary Table 1).

Prevalence of T-LGL was higher in Jeonbuk-state (18.65), Jeonnam-state (16.50) and Gwangju-city (15.37) (Fig. 5b). No associations of prevalence with number of medical specialists by region were found (data not shown).

## Discussion

This study demonstrated that, in Korea, the average annual crude incidence of PRCA is 2.77 per million, which is higher than Japan (1.06), but lower than a previous Korean study (5.28) [1, 3]. The previous Korean study [3] found that the annual crude incidence was higher at the beginning of the analysis year. This discrepancy may be due to the accumulation of data at the beginning of analysis year for PRCA prior to the year of data extraction. This study analyzed 20 years of PRCA crude incidence and found that the annual crude incidence did not change largely during that period. In contrast, T-LGL incidence during the 2011–2022 was more than twice as high as in 2003–2010, and previous study had also shown a 13.5-fold increase in incidence in 2015–2019 compared to 2000–2005 [8]. The increase of T-LGL incidence may be attributed to greater diagnostic familiarity of clinicians through laboratory testing, including T-cell receptor gene rearrangement [9] and next generation sequencing to detect *STAT3* mutations, which are often detected in T-LGL [10]. 7–36% of T-LGL are diagnosed even with a low large granular lymphocyte count (0.5-1.0 × 10^9^/L) in peripheral blood, and the potential for T-LGL in these patients may have been underestimated in the 2000s [11].

Many studies have reported predominance of PRCA in females and have speculated on autoimmune disease and sex hormone as contributing factors [1, 3]. In this study, the female predominance persisted during the 20 years, but the median female-to-male ratio of PRCA decreased from 1.82 between 2013 and 2022 to 1.31 between 2003 and 2012. As Korea becomes an aged society and anemia is more common in elderly, and the increasing number of elderly males in the population [6]. T-LGL incidence did not differ by gender, which confirms similar results from previous studies [12, 13]. The shift to an aged society is also thought to have affected the age at diagnosis of PRCA. In Korea, the median age at diagnosis of PRCA was 45–58 years in the 2000s, but increased to 60–67 years in the 2010s. In Japan, which has entered a super-aged society, the mean age was 55 years in 2000s [14], and increased to 73 years in the 2010s [1].

In this study, the most common condition associated PRCA was rheumatic diseases (10.5%), a higher rate than the approximately 3% reported in previous systematic review [15]. This discrepancy may be attributed to the inclusion of patients with prior medical histories over 20 year analysis period, which may have led to higher estimates compared to previous reports.

This suggests that the immune mechanism is strongly associated with PRCA, and supports previous studies that PRCA is caused by an immune-mediated erythropoietic failure [16, 17]. In this study, the frequencies of thymoma (4.7%) and T-LGL (0.6%) were lower than those reported in a Japanese study [1], which reported thymoma (9.4%) and large granular lymphocyte leukemia (2.5%) as the main causes of PRCA, with the majority of cases being idiopathic (69.0%). Autoimmune diseases were not reported. The proportion of thymoma associated with PRCA in both studies was similar to the 5–10% reported in previous studies [16, 18].

To our knowledge, this is the first study to investigate the sequential events of PRCA and T-LGL onset in patients with both PRCA and T-LGL. Among a total of 18 patients having both PRCA and T-LGL, 9 (50.0%) patients exhibited PRCA followed by T-LGL, while 1 (5.6%) patient exhibiting the opposite sequence where T-LGL was followed by PRCA. This result raises the possibility that, PRCA is primarily caused by autoreactive T cells which suppress erythropoiesis and sequentially evolve into clonal T cell proliferation, with the eventual occurrence of T-LGL [16]. During T cell clonal expansion, *STAT3* mutation may occur, causing survival advantage and finally resulting in T-LGL development. It is interesting that *STAT3* mutations commonly detected in T-LGL patients have also been reported in 43% of PRCA cases, implying clonal T cell proliferation in PRCA [19, 20]. Our prior study [7] showed that patients with PRCA combined with T-LGL had low VAF level of *STAT3* mutations compared with those with T-LGL alone. This indicates that PRCA combined with T-LGL is a unique disease entity from T-LGL alone, as low VAF *STAT3* mutation represents newly developing T-LGL clone in the background condition of PRCA. Further study on *STAT3* mutation status is required to identify the actual causal relationship.

Additionally, 8 out of 18 patients (44.4%) exhibited concurrent diagnoses of PRCA and T-LGL. The higher frequency of PRCA in *STAT3* mutated T-LGL than in *STAT3* unmutated T-LGL suggests common pathogenic mechanism between PRCA and T-LGL [21]. This suggests *STAT3*-related deregulation and activation of *STAT3* is possible major contributor not only to T-LGL but also to PRCA. From a therapeutic perspective, PRCA responds well to immunosuppressive agents like cyclosporin A [2] may affect the treatment of subclonal T-LGL.

The prevalence of both diseases varied by region, and a temporary high incidence of PRCA in Chungbuk-state from 2011 to 2016 may have contributed to the high prevalence. This may have been due to improved diagnostic capabilities at local healthcare facilities, and an outflow of younger people when Sejong-city was built as a new city in 2012 may have affected the demographics and prevalence of Chungbuk-state. Other possible factors include environmental factors, discrepancies between ICD-10 coding by medical specialists, and human leukocyte antigen diversity [22, 23]. These factors could be considered for further investigation in future studies.

## Conclusion

This study, using NHIS-NHID data, showed that the average annual crude incidence of PRCA was 2.77 per million with no significant change over 20 years, while T-LGL had an incidence of 0.82 per million and showed increasing trends, likely due to improved diagnostic testing. The median ages of onset for both PRCA and T-LGL increased over times, reflecting aging society. Rheumatic diseases were most common comorbidity in PRCA, highlighting the role of immune mechanisms. Notably, in patients with both PRCA combined with T-LGL, PRCA generally preceded T-LGL or diagnosed concurrently, suggesting autoreactive T cell proliferation in the background PRCA may drive the subsequent development of T-LGL, and both conditions might share a common pathogenic pathway. Further research into *STAT3* mutation status is expected to identify the causal relationship of between PRCA and T-LGL.

### Limitations

We identified some limitations to the scope of our study. Heterogeneity of immunophenotype, mutational profile and treatment response could not be evaluated as laboratory results were not registered in the NHIS-NHID. In the early years of the analysis, the number of patients could be overstated due to the short wash-out period of the big data. Due to the wide variations in the associated conditions of T-LGL, they could not be evaluated.

## Electronic supplementary material

Below is the link to the electronic supplementary material.

Supplementary Material 1

## Data Availability

This study used National Health Insurance Service (NHIS)-National Health Information Database, which the authors do not have permission to share. Requests to access the data should be directed to NHIS.
