# Supplementary material for: Epidemiologic sequential analysis of pure red blood cell aplasia and T-cell large granular lymphocyte leukemia in Korea
Source: Ann Hematol. 2025 May 22;104(5):2881–7. doi: 10.1007/s00277-025-06406-x (PMC12141113; doi:10.1007/s00277-025-06406-x)
Supplement: Supplementary file 1 — Supplementary Material 1 [file 277_2025_6406_MOESM1_ESM.docx]

**Epidemiologic sequential analysis of pure red blood cell aplasia and T-cell large granular lymphocyte leukemia in Korea**

Sooyong Park^1^, Hyun Kyung Kim^2^

^1^Department of Laboratory Medicine, Chungnam National University Hospital, Daejeon, Republic of Korea, ^2^Department of Laboratory Medicine, Seoul National University College of Medicine, Seoul, Republic of Korea

**Supplementary Table. 1** Annual incidence of pure red cell aplasia by region

| Region | Year | | | | | | | | | | | | | | | | | | | |
| --- | --- | --- | --- | --- | --- | --- | --- | --- | --- | --- | --- | --- | --- | --- | --- | --- | --- | --- | --- | --- |
|  | 2003 | 2004 | 2005 | 2006 | 2007 | 2008 | 2009 | 2010 | 2011 | 2012 | 2013 | 2014 | 2015 | 2016 | 2017 | 2018 | 2019 | 2020 | 2021 | 2022 |
| Seoul-city | 3.34 | 4.13 | 2.75 | 1.67 | 3.43 | 1.67 | 3.13 | 1.75 | 2.05 | 1.57 | 3.25 | 2.57 | 2.00 | 2.82 | 3.65 | 2.36 | 2.98 | 2.59 | 2.84 | 2.97 |
| Busan-city | 4.61 | 2.18 | 2.20 | 0.83 | 3.07 | 3.65 | 2.26 | 2.80 | 3.38 | 1.98 | 3.97 | 3.41 | 2.85 | 3.72 | 2.59 | 3.78 | 2.64 | 2.36 | 3.58 | 3.62 |
| Daegu-city | 5.53 | 1.98 | 3.19 | 2.40 | 2.41 | 2.01 | 3.61 | 0.80 | 3.59 | 3.19 | 2.00 | 2.01 | 2.81 | 3.22 | 1.21 | 0.41 | 3.28 | 4.55 | 1.26 | 2.96 |
| Incheon-city | 5.06 | 5.82 | 1.54 | 1.91 | 0.75 | 2.60 | 2.58 | 0.73 | 2.14 | 3.52 | 2.43 | 2.76 | 2.05 | 2.72 | 3.05 | 3.05 | 3.72 | 3.40 | 3.39 | 2.70 |
| Gwangju-city | 2.15 | 4.28 | 1.43 | 3.55 | 3.54 | 3.51 | 4.19 | 1.37 | 2.05 | 2.72 | 2.72 | 4.74 | 4.08 | 3.40 | 2.73 | 0.00 | 2.06 | 2.07 | 2.08 | 2.80 |
| Daejeon-city | 4.19 | 2.77 | 5.50 | 2.73 | 2.71 | 1.35 | 2.70 | 3.99 | 2.64 | 1.97 | 2.61 | 3.26 | 2.63 | 1.32 | 3.33 | 2.68 | 2.71 | 4.10 | 6.20 | 6.22 |
| Ulsan-city | 3.73 | 0.92 | 2.76 | 1.83 | 0.91 | 2.70 | 0.90 | 1.78 | 2.64 | 2.61 | 1.73 | 0.00 | 0.85 | 0.85 | 2.57 | 2.60 | 5.23 | 2.64 | 1.78 | 0.90 |
| Sejong-city | N/A^*^ | N/A | N/A | N/A | N/A | N/A | N/A | N/A | N/A | 0.00 | 8.19 | 0.00 | 0.00 | 0.00 | 0.00 | 0.00 | 5.87 | 0.00 | 2.69 | 7.82 |
| Gyeonggi-state | 3.23 | 3.82 | 2.80 | 1.93 | 3.15 | 2.30 | 1.66 | 1.78 | 2.09 | 1.07 | 1.55 | 1.62 | 2.16 | 2.36 | 1.17 | 1.68 | 1.96 | 1.94 | 1.92 | 2.72 |
| Gangwon-state | 2.62 | 3.29 | 1.32 | 3.32 | 7.98 | 1.99 | 1.32 | 2.61 | 3.91 | 3.25 | 7.13 | 1.29 | 4.52 | 3.87 | 0.65 | 1.94 | 3.24 | 1.94 | 2.60 | 1.30 |
| Chungbuk-state | 3.36 | 2.69 | 6.05 | 4.68 | 5.97 | 2.63 | 3.27 | 3.23 | 8.96 | 9.58 | 5.09 | 10.77 | 13.89 | 10.05 | 6.27 | 4.38 | 5.00 | 5.62 | 6.89 | 6.27 |
| Chungnam-state | 3.66 | 2.56 | 3.57 | 2.03 | 1.50 | 4.95 | 1.47 | 2.89 | 3.33 | 3.45 | 1.95 | 1.94 | 4.81 | 2.86 | 2.36 | 1.41 | 2.35 | 2.36 | 4.72 | 6.59 |
| Jeonbuk-state | 2.05 | 5.77 | 4.77 | 2.14 | 4.83 | 2.69 | 4.31 | 2.68 | 4.27 | 4.27 | 4.27 | 2.67 | 1.60 | 2.68 | 3.24 | 5.99 | 2.75 | 1.66 | 3.92 | 3.39 |
| Jeonnam-state | 2.97 | 1.51 | 1.53 | 2.06 | 3.63 | 3.13 | 1.57 | 4.17 | 3.13 | 4.19 | 2.10 | 5.77 | 4.71 | 5.78 | 1.05 | 3.19 | 4.28 | 4.32 | 2.73 | 3.85 |
| Gyeongbuk-state | 3.31 | 2.23 | 5.21 | 1.49 | 2.24 | 3.74 | 2.62 | 2.23 | 1.85 | 1.48 | 2.96 | 2.59 | 3.33 | 1.48 | 1.86 | 2.24 | 4.13 | 0.76 | 3.81 | 2.69 |
| Gyeongnam-state | 3.82 | 3.50 | 4.43 | 2.84 | 2.82 | 3.72 | 3.08 | 2.43 | 2.12 | 2.71 | 3.00 | 1.79 | 1.19 | 1.19 | 2.66 | 1.19 | 2.38 | 2.69 | 3.62 | 2.44 |
| Jeju-state | 0.00 | 3.60 | 1.79 | 3.58 | 1.79 | 0.00 | 7.11 | 3.50 | 6.94 | 6.85 | 8.42 | 4.94 | 1.60 | 1.56 | 6.09 | 6.00 | 2.98 | 1.48 | 4.43 | 2.95 |
| Total | 3.53 | 3.46 | 3.07 | 2.08 | 3.15 | 2.58 | 2.57 | 2.12 | 2.76 | 2.43 | 2.87 | 2.69 | 2.83 | 2.86 | 2.43 | 2.30 | 2.89 | 2.55 | 3.00 | 3.21 |

Numbers are incidence (per million per year). Abbreviation: N/A, not available
^*^Sejong-City was newly established on July 1, 2012, as a result of a change in administrative region, so incidence prior to this day was not available

**Supplementary Table. 2** Annual incidence of T-cell large granular leukemia by region

| Region | Year | | | | | | | | | | | | | | | | | | | |
| --- | --- | --- | --- | --- | --- | --- | --- | --- | --- | --- | --- | --- | --- | --- | --- | --- | --- | --- | --- | --- |
|  | 2003 | 2004 | 2005 | 2006 | 2007 | 2008 | 2009 | 2010 | 2011 | 2012 | 2013 | 2014 | 2015 | 2016 | 2017 | 2018 | 2019 | 2020 | 2021 | 2022 |
| Seoul-city | 0.10 | 0.20 | 0.20 | 0.79 | 0.59 | 0.20 | 0.49 | 0.29 | 1.37 | 0.88 | 0.49 | 1.09 | 1.10 | 1.51 | 1.12 | 1.54 | 0.62 | 0.93 | 1.58 | 1.17 |
| Busan-city | 0.00 | 0.55 | 0.00 | 0.00 | 0.28 | 0.28 | 0.00 | 0.00 | 1.13 | 0.85 | 0.57 | 0.28 | 0.57 | 0.57 | 0.86 | 2.03 | 0.88 | 0.88 | 0.00 | 1.21 |
| Daegu-city | 0.00 | 0.40 | 0.40 | 0.40 | 0.00 | 0.80 | 0.00 | 0.40 | 0.40 | 1.20 | 0.40 | 1.20 | 0.80 | 0.00 | 0.40 | 0.00 | 0.82 | 0.00 | 0.00 | 1.27 |
| Incheon-city | 0.39 | 0.00 | 0.00 | 0.76 | 0.75 | 1.11 | 0.00 | 0.00 | 0.36 | 0.35 | 0.69 | 0.00 | 0.68 | 1.02 | 0.68 | 0.34 | 1.01 | 0.68 | 0.68 | 1.01 |
| Gwangju-city | 0.72 | 0.71 | 0.00 | 0.71 | 1.41 | 1.41 | 1.40 | 0.00 | 0.00 | 3.40 | 4.75 | 6.78 | 0.68 | 2.04 | 2.05 | 2.06 | 0.69 | 1.38 | 2.08 | 0.00 |
| Daejeon-city | 0.00 | 0.00 | 0.69 | 0.00 | 2.03 | 0.00 | 0.67 | 0.67 | 0.66 | 1.31 | 1.30 | 1.96 | 0.00 | 0.00 | 1.33 | 0.00 | 0.00 | 0.68 | 2.75 | 1.38 |
| Ulsan-city | 0.00 | 0.00 | 0.00 | 0.92 | 0.00 | 0.90 | 0.90 | 0.89 | 0.88 | 0.00 | 0.00 | 0.00 | 1.70 | 0.00 | 0.00 | 0.00 | 0.00 | 0.00 | 0.00 | 0.00 |
| Sejong-city | N/A^*^ | N/A | N/A | N/A | N/A | N/A | N/A | N/A | N/A | 0.00 | 0.00 | 0.00 | 0.00 | 0.00 | 3.57 | 0.00 | 2.94 | 2.81 | 0.00 | 2.61 |
| Gyeonggi-state | 0.10 | 0.10 | 0.28 | 0.28 | 0.45 | 0.35 | 0.26 | 0.68 | 1.01 | 0.83 | 0.74 | 0.57 | 1.28 | 1.02 | 1.17 | 0.84 | 0.68 | 1.12 | 1.70 | 1.10 |
| Gangwon-state | 0.00 | 0.00 | 0.00 | 1.33 | 0.66 | 0.00 | 1.98 | 0.65 | 0.00 | 0.65 | 0.00 | 1.94 | 0.65 | 0.64 | 0.65 | 0.00 | 1.95 | 0.00 | 1.30 | 0.65 |
| Chungbuk-state | 0.67 | 0.00 | 0.00 | 0.00 | 1.33 | 0.66 | 0.00 | 0.00 | 0.64 | 0.64 | 1.27 | 2.53 | 1.89 | 2.51 | 0.63 | 0.00 | 1.87 | 0.62 | 0.63 | 1.25 |
| Chungnam-state | 0.52 | 0.51 | 0.00 | 0.51 | 1.50 | 0.00 | 0.49 | 1.45 | 0.95 | 0.49 | 0.98 | 0.97 | 1.93 | 0.48 | 1.42 | 0.94 | 1.88 | 1.89 | 1.42 | 0.47 |
| Jeonbuk-state | 0.00 | 0.52 | 1.59 | 0.00 | 1.07 | 0.00 | 2.70 | 1.61 | 1.07 | 0.00 | 0.53 | 0.53 | 2.67 | 3.22 | 2.16 | 2.18 | 4.95 | 1.66 | 1.68 | 2.83 |
| Jeonnam-state | 0.99 | 0.00 | 0.00 | 1.54 | 0.00 | 2.08 | 0.52 | 0.52 | 3.13 | 6.81 | 3.15 | 2.62 | 2.10 | 5.78 | 2.11 | 3.19 | 2.14 | 1.62 | 2.18 | 1.65 |
| Gyeongbuk-state | 0.37 | 0.00 | 0.37 | 1.12 | 0.00 | 1.12 | 0.00 | 0.74 | 1.48 | 0.00 | 0.74 | 1.85 | 1.48 | 2.22 | 0.74 | 0.75 | 1.50 | 0.76 | 1.90 | 1.54 |
| Gyeongnam-state | 0.00 | 0.32 | 0.00 | 0.00 | 0.31 | 0.00 | 1.23 | 0.30 | 0.60 | 0.90 | 1.20 | 0.30 | 0.59 | 0.30 | 0.89 | 1.48 | 0.00 | 0.90 | 2.11 | 0.61 |
| Jeju-state | 0.00 | 0.00 | 0.00 | 0.00 | 0.00 | 0.00 | 0.00 | 1.75 | 3.47 | 0.00 | 1.68 | 1.65 | 0.00 | 3.12 | 1.52 | 0.00 | 1.49 | 1.48 | 1.48 | 0.00 |
| Total | 0.19 | 0.21 | 0.23 | 0.51 | 0.57 | 0.46 | 0.52 | 0.51 | 1.04 | 1.02 | 0.90 | 1.11 | 1.14 | 1.32 | 1.10 | 1.08 | 1.02 | 0.96 | 1.41 | 1.11 |

Numbers are incidence (per million per year). Abbreviation: N/A, not available
^*^Sejong-City was newly established on July 1, 2012, as a result of a change in administrative region, so incidence prior to this day was not available

**Supplementary Table. 3** Prevalence of PRCA and T-LGL by region at December 31, 2022

| Region | Population (N) | PRCA | | T-LGL | |
| --- | --- | --- | --- | --- | --- |
|  |  | Patient (N) | Prevalence^*^ | Patient (N) | Prevalence^*^ |
| Seoul-city | 9,428,372 | 350 | 37.12 | 101 | 10.71 |
| Busan-city | 3,317,812 | 120 | 36.17 | 20 | 6.03 |
| Daegu-city | 2,363,691 | 77 | 32.58 | 11 | 4.65 |
| Incheon-city | 2,967,314 | 101 | 34.04 | 19 | 6.40 |
| Gwangju-city | 1,431,050 | 45 | 31.45 | 22 | 15.37 |
| Daejeon-city | 1,446,072 | 69 | 47.72 | 13 | 8.99 |
| Ulsan-city | 1,110,663 | 29 | 26.11 | 3 | 2.70 |
| Sejong-city | 383,591 | 5 | 13.03 | 3 | 7.82 |
| Gyeonggi-state | 13,589,432 | 327 | 24.06 | 126 | 9.27 |
| Gangwon-state | 1,536,498 | 59 | 38.40 | 8 | 5.21 |
| Chungbuk-state | 1,595,058 | 130 | 81.50 | 13 | 8.15 |
| Chungnam-state | 2,123,037 | 76 | 35.80 | 18 | 8.48 |
| Jeonbuk-state | 1,769,607 | 88 | 49.73 | 33 | 18.65 |
| Jeonnam-state | 1,817,697 | 76 | 41.81 | 30 | 16.50 |
| Gyeongbuk-state | 2,600,492 | 79 | 30.38 | 32 | 12.31 |
| Gyeongnam-state | 3,280,493 | 106 | 32.31 | 20 | 6.10 |
| Jeju-state | 678,159 | 26 | 38.34 | 6 | 8.85 |
| Total | 51,439,038 | 1,763 | 34.27 | 478 | 9.29 |

Abbreviations: PRCA, pure red cell aplasia; T-LGL, T-cell large granular leukemia
^*^Prevalence: Numbers are prevalence (per million)
